# Genome Analysis of *Endobacterium cerealis*, a Novel Genus and Species Isolated from *Zea mays* Roots in North Spain

**DOI:** 10.3390/microorganisms8060939

**Published:** 2020-06-22

**Authors:** Esther Menéndez, Jose David Flores-Félix, Martha Helena Ramírez-Bahena, Jose M. Igual, Paula García-Fraile, Alvaro Peix, Encarna Velázquez

**Affiliations:** 1 Mediterranean Institute for Agriculture, Environment and Development (MED), Institute for Advanced Studies and Research (IIFA), Universidade de Évora, Pólo da Mitra, Ap. 94, 7006-554 Évora, Portugal; esthermenendez@uevora.pt; 2 Departamento de Microbiología y Genética and Instituto Hispanoluso de Investigaciones Agrarias (CIALE), Universidad de Salamanca, 37007 Salamanca, Spain; jdflores@usal.es (J.D.F.-F.); paulagf81@usal.es (P.G.-F.); evp@usal.es (E.V.); 3 Instituto de Recursos Naturales y Agrobiología, IRNASA-CSIC, 37008 Salamanca, Spain; marthah.ramirez@irnasa.csic.es (M.H.R.-B.); mariano.igual@irnasa.csic.es (J.M.I.); 4 Unidad Asociada Grupo de Interacción Planta-Microorganismo, Universidad de Salamanca-IRNASA-CSIC, 37008 Salamanca, Spain

**Keywords:** *Endobacterium* gen. nov., *Endobacterium cerealis* sp. nov., *Zea mays*, Spain, endophytes, genome analysis

## Abstract

In the present work, we analyse the genomic and phenotypic characteristics of a strain named RZME27^T^ isolated from roots of a *Zea mays* plant grown in Spain. The phylogenetic analyses of 16S rRNA gene and whole genome sequences showed that the strain RZME27^T^ clustered with the type strains of *Neorhizobium galegae* and *Pseudorhizobium pelagicum* from the family *Rhizobiaceae*. This family encompasses several genera establishing symbiosis with legumes, but the genes involved in nodulation and nitrogen fixation are absent in its genome. Nevertheless, genes related to plant colonization, such as those involved in motility, chemotaxis, quorum sensing, exopolysaccharide biosynthesis and hydrolytic enzymes production were found. The comparative pangenomic analyses showed that 78 protein clusters present in the strain RZME27^T^ were not found in the type strains of its closest relatives *N. galegae* and *P. pelagicum*. The calculated average nucleotide identity (ANI) values between the strain RZME27^T^ and the type strains of *N. galegae* and *P. pelagicum* were 75.61% and 75.1%, respectively, similar or lower than those found for other genera from family *Rhizobiaceae*. Several phenotypic differences were also found, highlighting the absence of the fatty acid C_19:0_ cyclo ω8c and propionate assimilation. These results support the definition of a novel genus and species named *Endobacterium cerealis* gen. nov. sp. nov. whose type strain is RZME27^T^.

## 1. Introduction

The family *Rhizobiaceae* [1] belongs to the class Alphaproteobacteria and currently, this family comprises the classical genera *Rhizobium* [2], *Agrobacterium* [3], *Sinorhizobium* [4] (later transferred to *Ensifer* [5]), and *Allorhizobium* [6] as well as the more recently described genera *Shinella* [7], *Ciceribacter* [8], *Neorhizobium* [9], *Pararhizobium* [10], *Pseudorhizobium* [11], *Gellertiella* [12] and *Georhizobium* [13]. Several of these genera contain species originally isolated from legume nodules, such as *Rhizobium*, *Ensifer* (*Sinorhizobium*), *Allorhizobium*, *Shinella*, *Neorhizobium* or *Pararhizobium* [14,15,16] and from plant tumours such as *Agrobacterium* [17]. However, other genera within this family have not been reported to date as legume endosymbionts, plant endophytes or plant pathogens, such as *Ciceribacter*, *Pseudorhizobium*, *Gellertiella* and *Georhizobium* [11,12,13]. Some genera contain species originally isolated from cereals, but to date only two species of the genus *Rhizobium*, *Rhizobium zeae* [18] and *Rhizobium wenxiniae* [19], have been isolated from *Zea mays* (maize) roots.

The endophytic bacterial community of maize has been widely studied and several new endophytic species of this cereal have been described, but only two new genera, *Dyadobacter* [20] and *Runella* [21], were isolated from stems of *Zea mays* and they belong to the family *Cytophagaceae* within the Class Flavobacteria. In this work, we report the existence of a novel genus belonging to the family *Rhizobiaceae*, which was isolated from roots of *Zea mays* growing in a field of León province (Northern Spain), the largest and most productive area for maize cropping in Spain. The aims of this study were the genome analysis of this novel genus for which we proposed the name *Endobacterium* gen. nov. and the characterization through the analysis of genomic, chemotaxonomic and phenotypic traits of its first described species *Endobacterium cerealis* gen. nov. sp. nov., whose type strain, RZME27^T^, has been deposited in two international culture collections under the accession numbers LMG 31256^T^ and CECT 9794^T^.

## 2. Materials and Methods

### 2.1. Strain Isolation

The strain RZME27^T^ was isolated from roots of a *Zea mays* plant harvested six months after the sowing in a field in Riego de la Vega (León, NW Spain, 42°23′21″ N 5°58′56″ O). The roots were surface disinfected with 70% ethanol for 1 min, 2% NaClO for 3 min and 70% ethanol for 30 s. Then, the roots were rinsed five times with sterile distilled water and crushed into a tube containing 10 mL of sterile phosphate-buffered saline (PBS) at pH7, which was further maintained in shaking at 160 rpm during 1 h at room temperature. Then, six serial decimal dilutions were obtained and 100 µL aliquots were spread onto Tryptic Soy Agar (TSA; BD Difco, Franklin Lakes, NJ, USA) plates and incubated for 48 h at 28 °C. In the higher dilution, a mucoid colony was picked and transferred to a new TSA plate and the isolated strain was named RZME27^T^. In parallel, in order to ensure their complete external disinfection, some of the surface-disinfected root samples were incubated in the same medium, showing no bacterial growth around them.

### 2.2. 16S rRNA Gene Phylogenetic Analysis

The 16S rRNA gene of strain RZME27^T^ was amplified and sequenced as previously described [22] at the Sequencing DNA Service of NUCLEUS, University of Salamanca (Spain). The 16S rRNA gene sequence obtained was compared with those held in GenBank [23] and for its phylogenetic analysis, the sequence was aligned with those of the type strains of closest-related species using the ClustalW program [24]. The phylogenetic distances were calculated according to Kimura’s two-parameter model [25]. The phylogenetic trees were inferred using the neighbour joining (NJ) and maximum likelihood (ML) algorithms [26,27] and MEGA 7.09 [28] was used for all phylogenetic analyses.

### 2.3. Genome Sequencing, Assembling, Annotation and Analysis

The genomic DNA from a pure culture of the strain RZME27^T^ was purified using the DNeasy UltraClean Microbial DNA Isolation Kit (Qiagen, Venlo, Netherlands) following manufacturer’s protocol. Sequencing, upon preparation of pair-end libraries, was performed on Illumina MiSeq sequencing platform (2 × 250 bp). Sequencing data was assembled using Velvet 1.12.10 [29]. Annotation was undertaken using RAST 2.0 (Rapid Annotation using Subsystem Technology) [30,31] and the NCBI (National Center for Biotechnology Information) Prokaryotic Genome Annotation Pipeline (PGAP) (https://www.ncbi.nlm.nih.gov/genome/annotation_prok/) [32,33]. The draft genome sequence of the strain RZME27^T^ was deposited in DDBJ/EMBL/GenBank under the BioProject PRJNA576662 (accession number WIXI00000000). Data from the type strains of related species and genera were retrieved from public databases (Table S1). The circular genome map of the strain RZME27^T^ and the schematic map of BLAST (Basic Local Alignment Search Tool) comparison among closest type strain genomes was generated using the CGView server (http://cgview.ca/; accessed 17th March 2020) [34,35]. Phylogenomic analysis was conducted with the Type Strain Genome Server [36]. This web-server tool employs the Genome-BLAST Distance Phylogeny method (GBDP) [37] to compare whole genome sequences at nucleotide level, allowing to calculate dDDH value and construct the phylogram. The Average Nucleotide Identity using BLAST (ANIb) value was calculated with JSpeciesWS Online Service, which is based on BLAST+ (version 2.2.29+) [38]. Carbohydrate-active enzyme analysis was performed using the dbCAN2 meta server (http://cys.bios.niu.edu/dbCAN2; accessed 22th March 2020) [39]. Secondary metabolite production potential was carried out analyzing the presence in the genome of biosynthetic gene clusters (BGCs) within the tool antiSMASH version 5.1.1 [40]. KofamKOALA tool was used to annotate the genome based on the KEGG (Kyotto Encyclopedia of Genes and Genomes) database [41] Comparative genome-wide analysis of orthologous clusters and gene ontology analysis among all predicted protein-coding genes was performed using OrthoVenn2 (https://orthovenn2.bioinfotoolkits.net/home; accessed 20th March 2020) [42]. The genomes of phylogenetically related strains were retrieved from GenBank (https://www.ncbi.nlm.nih.gov/genome/; accessed 15th March 2020) (Supplementary Table S1).

### 2.4. Fatty Acid Analysis and Phenotypic Characterization

The cellular fatty acids were analysed by using the Microbial Identification System (MIDI; Microbial ID) Sherlock 6.1 and the library RTSBA6 according to the technical instructions provided by this system [43]. The strains RZME27^T^, *Neorhizobium galegae* HAMBI 540^T^, *Pseudorhizobium pelagicum* R1-200B4^T^ and *Rhizobium leguminosarum* USDA 2370^T^ were cultured aerobically on Tryptone Yeast agar (TY) plates [44] at 28 °C and cells were collected after 48 h incubation. Gram staining was carried out by the procedure described by Doetsch [45] after 24 h of incubation at 28 °C. The strain was grown on nutrient agar (NA; BD Difco, Franklin Lakes, NJ, USA) for 48 h at 22 °C to check for motility by phase-contrast microscopy using the hanging drop method. The flagellation type was determined by electron microscopy after 48 h of incubation of strain RZME27^T^ on Yeast Mannitol Agar (YMA; Laboratorios Microkit, Madrid, Spain) plates at 22 °C. The cells were gently suspended in sterile water and then stained with 2% uranyl acetate and examined at 80 kV with a JEOL 1010 transmission electron microscope equipped with a Gatan Bioscan 792 digital camera from the Microscopy Service of NUCLEUS, University of Salamanca (Spain). The phenotypic characterization was performed in this study using the API ID32GN and API 20NE systems (BioMérieux, Marcv L’Etoile, France) in the conditions indicated by the manufacturer and the results were read after 48 h of incubation at 28°C. Growth temperature range was determined by incubating cultures in YMA medium [46] at 4, 15, 28, 37 and 45 °C. Growth pH range was determined in the same medium with final pH 5, 6, 7, 8 and 9. PCA buffer (phosphate citric acid buffer; Na_2_HPO_4_ 0.4 M and citric acid 0.2 M) was used to adjust the pH from 5 and 6, phosphate buffer (Na_2_HPO_4_ 0.2 M and NaH_2_PO_4_ 0.2 M) was used for pH 7 and TE buffer 0.2 M was used for pH 8 and 9. Salt tolerance was tested in the same medium containing 0.5, 1, 1.5, 2, 2.5, 4 and 7% (*w/v*) NaCl. Catalase production was assayed by using 0.3% hydrogen peroxide with one colony taken from the TY plates. Oxidase activity was detected by using *N*,*N*,*N*′,*N*′-tetramethyl-1,4-phenylenediamine dihydrochloride. The strains *Neorhizobium galegae* HAMBI 540^T^, *Pseudorhizobium pelagicum* R1-200B4^T^ and *Rhizobium leguminosarum* USDA 2370^T^ were used as reference

## 3. Results

### 3.1. 16S rRNA Gene Phylogenetic Analysis

**Figure 1:**
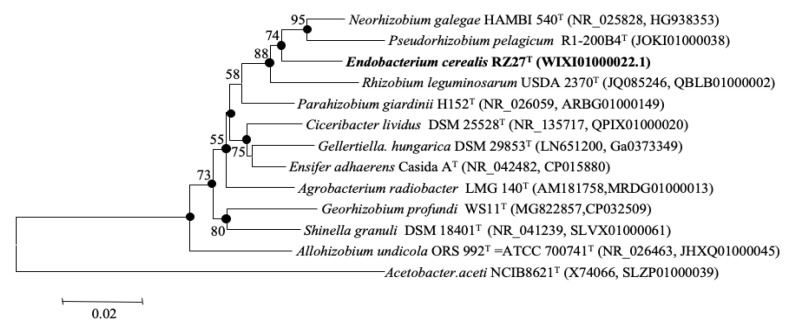
Neighbour Joining phylogenetic tree based on 16S rRNA gene sequences (1433 nt) showing the taxonomic location of the type strain of *Endobacterium cerealis* within the family *Rhizobiaceae*. Bootstrap values calculated for 1000 replications are indicated. Bar, 1 nt substitution per 1000 nt. Accession numbers from GenBank are given in brackets. The nodes marked with filled circles were also obtained with the maximum likelihood algorithm.

The comparison against GenBank of the 16S rRNA gene showed that the strain RZME27^T^ belongs to the family *Rhizobiaceae* and that it is equidistant to different species of genera *Neorhizobium* and *Rhizobium*, showing similarity values ranging from 97% to 98%. The type strains of the type species of these genera, *N. galegae*, and *R. leguminosarum*, showed 97.4% and 95.8% similarity, respectively, with respect to the strain RZME27^T^. Similarity values higher than 95% were also presented by the type strains of *P. pelagicum*, *Agrobacterium radiobacter* and *Pararhizobium giardinii* (Supplementary Table S2). Similarity values equal or lower than 95% were found for the type strains of the type species of the remaining genera of the family *Rhizobiaceae* (Supplementary Table S2). Similarity values higher than 97% also were found between the type species of *Pseudorhizobium* and *Neorhizobium* and between the type species of *Ensifer* and those of *Ciceribacter*, *Gellertiella* and *Pararhizobium* (Supplementary Table S2). The NJ and ML phylogenetic analyses of the 16S rRNA gene showed that the new strain RZME27^T^ clustered with the type strains of *N. galegae*, *P. pelagicum* and *R. leguminosarum* (Figure 1).

### 3.2. Genome Analyses

#### 3.2.1. Genome Properties

**Figure 2:**
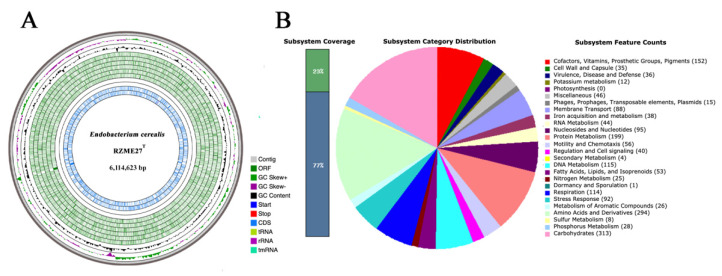
Genome circular representation and subsystems category distribution of annotated genes of the *Endobacterium cerealis* RZME27^T^ genome. (**A**) From the outer circle to the inner circle, the genomic map of RZME27^T^ shows: contigs (light grey), open reading frames (ORFs; dark green), GC skew curves (+/−; green/purple), GC content (black), Start/Stop codons (dark blue/red), coding sequences (CDSs; blue), tRNAs (light green), rRNAs (violet) and tmRNAs (light blue). (**B**) Annotation of RZME27^T^ genome via the RAST server. The panel shows the annotation coverage, the categories distribution and the feature counts.

The draft genome of strain RZME27^T^ is composed by 51 contigs with a total genomic length of 6,114,623 bp and a G+C content of 58.80% (Table 1, Figure 2A). Genome annotation tools NCBI PGAP and RAST pipeline identified 5893 genes and 5836 codifying sequences, respectively (Table 1).

#### 3.2.2. Genome Mining

Using the RAST pipeline, we observed that genes are distributed into 25 subsystems according to SEED viewer in RAST (Supplementary Table S3, Figure 2B). However, this only represent the 23% of the genome, being the 77% of the genes unassigned to a subsystem. Nonetheless, we used manual mining to identify genes of interest within the genome of the strain RZME27^T^, even if these genes are not assigned to a subsystem. We identified genes involved in motility, chemotaxis and exopolysaccharide production, genes codifying degradative enzymes. However, genes related with siderophore production and symbiotic genes were not found in this genome.

Genes involved in pilus formation and in the type IV secretion system were found in the same genome subsystem within a genomic island. The genes involved in flagellar biosynthesis were found in a genome region containing genes codifying for flagellar biosynthesis proteins (FliR or FlhAB), flagellar basal-body rod proteins (FlgABCDFG) and flagellar motor rotor proteins (MotAB and FliMN). These genes are located in a specific region with chemotaxis genes (*che*) regulated by the *luxR* gene, which is a transcriptional regulator sensible to quorum sensing molecules. In addition, a *luxI* gene was located within a *luxR* gene in a quorum sensing operon in the strain RZME27^T^.

Several genes involved in exopolysaccharide biosynthesis are located in the genome of strain RZME27^T^, such as *exoD* and *exoZ*. In addition, other genes related with lipopolysaccharide biosynthesis, such as *wadC*, and lipopolysaccharide exportation, such as *lptABDFG*, have been annotated. Also, genes codifying other capsule formation related enzymes, such as those involved in poly-gamma-glutamate biosynthesis or exportation, were detected. Some genes involved in osmotic stress tolerance were also annotated with RAST, as choline dehydrogenase (EC.1.1.99.1) and betaine aldehyde dehydrogenase (EC.1.2.1.8), involved in the synthesis of the osmoprotectant glycinebetaine.

Using the dbCAN2 meta server, we identified a total of 73 genes encoding glycosyl hydrolases (GH) and 60 genes encoding glycosyl transferases (GT), amongst other carbohydrate-related enzymes and activities (Supplementary Table S4). The dbCAN2 meta server annotation also revealed the existence of 24 identified signal peptides.

The genome analysis reveals that this strain harbors three genes involved in cellulose biosynthesis related to those forming the *celABC* operon of *Rhizobium* strains. These genes, encoding for a cellulose synthase catalytic subunit [Uridine 5’-diphosphate(UDP)-forming] (EC 2.4.1.12), a cyclic di-GMP binding protein precursor and a ß-1,4-glucanase (cellulase) (EC 3.2.1.4), showed similarities of 79.84, 66.43 and 68.70% with the ones located in the genome of *Rhizobium etli* CFN42^T^.

Annotation using the KofamKOALA online tool also revealed the existence of complete metabolic pathways as well as many genes classified into pathways that are not complete (Supplementary Table S5). This annotation revealed the presence of high number of genes forming complete pathways related to carbohydrate metabolism. We also found genes potentially involved in the nitrate catabolism, but those genes are not forming a complete metabolic pathway. No genes involved in nodulation or nitrogen fixation were found using this annotation tool.

The potential production of secondary metabolites was scanned with antiSMASH, finding three possible gene clusters involved in the biosynthesis of a homoserine lactone, a terpene and a TfuA-related toxin, respectively. The first gene cluster shows between 100% and 63% similarity with those located in other strains from genus *Rhizobium* as *Rhizobium* sp. NFR07, *Rhizobium leucaenae* CPAO 29.8, *Rhizobium freirei* PRF 81^T^, *Rhizobium tropici* CIAT 899^T^ and *Rhizobium lusitanum* P1-7^T^. The cluster related with terpene biosynthesis also reveals around 80% similarity with gene clusters found in strains of *Neorhizobium galegae* and *Rhizobium* sp. On the contrary, the gene cluster predicted to be implicated in the synthesis of a TfuA-related toxin revealed only low similarity (12%) with gene clusters found in other members of the genera *Rhizobium* and *Ensifer*.

#### 3.2.3. Genetic Relatedness and Pangenome Analysis

**Figure 3:**
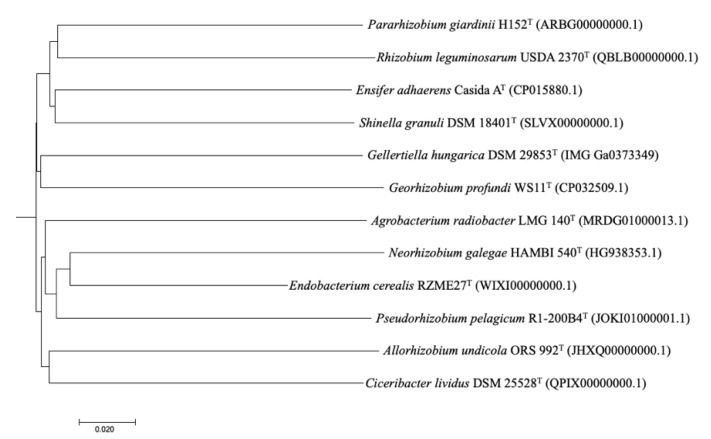
Whole genome based phylogenomic tree constructed with the Genome-BLAST Distance Phylogeny method (GBDP) tool and retrieved from the TYGS website. The tree was inferred with FastME 2.1.6.1 from GBDP distances calculated from genome sequences. The branch lengths are scaled in terms of GBDP distance formula *d*_5_.

The GBDP phylogenomic tree confirmed the phylogenetic position of the strain RZME27^T^ derived from the 16S rRNA gene analysis showing that this strain belongs to a cluster together the type strains of *N. galegae* HAMBI 540^T^ and *P. pelagicum* R1-200B4^T^ (Figure 3). The ANIb values between the whole genome of the strain RZME27^T^ and those of the type strains of the type species from the family *Rhizobiaceae* genera were lower than 76% (Table 2). The ANIb values between the strain RZME27^T^ and its closest relatives *N. galegae* and *P. pelagicum* were 75.6% and 75.1%, respectively. Similar or slightly higher values were found between the type species of *Ensifer* and *Pararhizobium*, *Pararhizobium* and *Shinella*, *Ensifer and Shinella* and *Pseudorhizobium* and *Neorhizobium* (Table 2). These results support the affiliation to the strain RZME27^T^ to a new genus within the family *Rhizobiaceae*.

**Figure 4:**
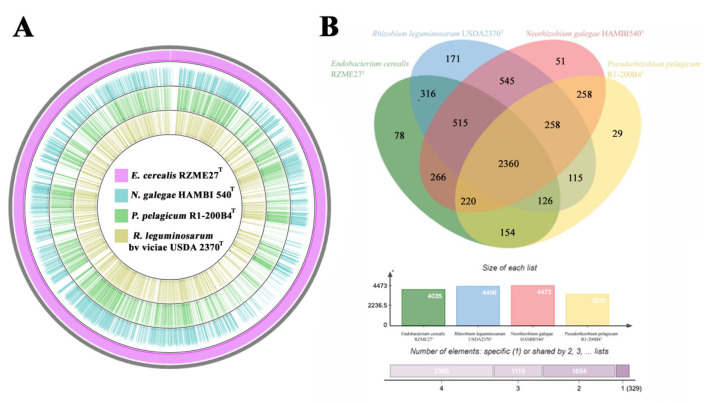
Comparative genomics among the genomes of *E. cerealis* RZME27^T^, *N. galegae* HAMBI 540^T^, *P. pelagicum* R1-200B4^T^ and *R. leguminosarum* USDA 2370^T^. (**A**) Schematic representation of BLAST comparisons amongst the four type species. From the outer to the inner circle: RZME27^T^ (pink), HAMBI 540^T^ (blue), R1-200B4^T^ (green) and USDA 2370^T^ (gold). (**B**) The Venn diagram and the bar chart below show the number of shared and unique orthologous amongst the four genomes.

The genomic comparison among the genomes of the strains *E. cerealis* RZME27^T^, *N. galegae* HAMBI 540^T^, *P. pelagicum* R1-200B4^T^ and *R. leguminosarum* USDA 2370^T^ is depicted in Figure 4A, showing that RZME27^T^ genome is closer to those of the type strains of *N. galegae* and *P. pelagicum* than to the genome of *R. leguminosarum* USDA 2370^T^.

The results of the comparative pan-genomic analysis are shown in a Venn diagram including the strain RZME27^T^ and its close related species (Figure 4B). The analysis shows a “core” genome composed by 2360 clusters of orthologous, most of them annotated as clusters of proteins with functions associated to cellular metabolic processes, motility, colonization and membrane exchange specialized systems. We found 78 protein clusters, which have been identified only in the strain RZME27^T^, whereas only 51 and 29 clusters were found, respectively, in its closest relative type strains of *P. pelagicum* and *N. galegae* (Figure 4B).

### 3.3. Fatty Acid Analysis and Phenotypic Characterization

The results of the fatty acid analysis are recorded in Table 3. The major fatty acids of the strain RZME27^T^ are those from summed feature 8 (C_18:1_ ω6c/C_18:1_ ω7c) and the C_16:0_ as occurs in its closest genera within family *Rhizobiaceae*. The absence of C_19:0_ cyclo ω8c in strain RZME27^T^ is the most relevant difference with respect to the rest of its related reference strains (Table 3).

The cells of strain RZME27^T^ are Gram negative straight rods and motile by means of one polar flagellum (Figure S1). The phenotypic characteristics of the strain RZME27^T^ are reported in the description of the new taxon and the differences with respect to its phylogenetic related genera are recorded in Table 4. The strain RZME27^T^ differs with respect to the type strain of *N. galegae* in the colonies colour, nitrate reduction and l-rhamnose assimilation, with respect to the type strain of *P. pelagicum* in assimilation of malate, inositol, melibiose, l-histidine and l-proline and with respect to the type strain of *R. leguminosarum* in nitrate reduction, growth in presence of 1% NaCl and at 37 °C, assimilation of 5-keto-gluconate and 3-hydroxybutyrate (Table 4).

## 4. Discussion

The family *Rhizobiaceae* mainly contains genera with strains nodulating legumes, but in the last years several of them have been isolated from sources different to the legume nodules [14,15,16]. This occurs in the case of the most recently described genera within the family, *Pseudorhizobium, Gellertiella* and *Georhizobium* isolated from water-related environments [11,12,13]. However, none of the *Rhizobiaceae* genera described to date were originally isolated from cereal related sources [11,12,13,14,15,16,17]. In this work we described the first new genus within family *Rhizobiaceae* isolated from roots of a cereal and whose type strain RZME27^T^ is to date the only available strain for this genus. Although, ideally the description of new taxa should be based on more than one strain [47], many recently described bacterial genera only contain the type strain, as occurs for example with the two most recently described genera in the family *Rhizobiaceae*, *Gellertiella* and *Georhizobium*, also isolated from sources other than legume nodules, whose descriptions were based on a single strain [12,13].

The definition of this new genus is based on the minimal standards for definition of new bacterial taxa which have been recently published [48] and include two successive steps, the first one based on the 16S rRNA gene analysis and the second one on the calculation of several parameters derived from genome analyses, known as OGRI [48]. From these parameters, the ANIb values have been taken as reference for genera differentiation, being the cut-off values previously established for those of the family *Rhizobiaceae* after the comparison of their genomes [11].

Following this scheme, we analysed the 16S rRNA gene of the strain RZME27^T^, which was phylogenetically equidistant to several species of genera *Rhizobium* and *Neorhizobium* (Supplementary Table S2) and the similarity values for this gene support the affiliation of this strain to a new genus since some of more recently proposed genus within the family *Rhizobiaceae* have similar or even higher similarity values in their 16S rRNA gene (Table 1). This occurs in the case of the genera *Pseudorhizobium* and *Neorhizobium* whose type species have 97.1% similarity and in the case of genera *Ciceribacter*, *Pararhizobium* and *Gellertiella* with respect to the genus *Ensifer*, with similarity values ranging from 97.5% to 97.7%. The description of all these genera was based on genetic analyses, which commonly included the phylogenies of the 16S rRNA and different housekeeping genes. Nevertheless, currently, the description of new taxa should be based on genome analyses and Chun et al. [48] proposed the minimal standards for the use of genome data for the taxonomy of Prokaryotes. Although they only give the ANI threshold values for species differentiation, they have been evaluated for genera delineation in different bacterial families. For example, in the family *Enterobacteriaceae* the different genera show ANI values ranging from 75 to 80% [49], including the recently described genus *Scandinavium* which has a ANIb value of 79.2% with respect to its closest related genus [50]. In the case of the Family *Rhizobiaceae*, it should be highlighted that the description of the genus *Pseudorhizobium* was based on genome analysis [11] and in this work the ANIb values among genomes of different genera belonging to the family *Rhizobiaceae* and to other families from the order *Rhizobiales* were analysed [11]. According to the results of this previous work, the current limit of ANIb value for genus delineation within several families of the order *Rhizobiales* was around 76% [11]. Recently, the description of the new genus *Georhizobium* was also supported by the results of ANIb calculation, with values lower than 76% with respect to the other genera of family *Rhizobiaceae* [13]. The ANIb values obtained for RZME27^T^ with respect to the type species of the genera included in the family *Rhizobiaceae* were lower than 76% in all cases, supporting the affiliation of this strain to a new genus within this family (Table 2).

Taking into account that this family contains several genera able establish nitrogen fixing symbiosis with legumes and that the new genus is related to *N. galegae*, which is able to induce effective nodules in *Galega* sp. [9], we searched for symbiotic genes involved in nodulation and nitrogen fixation, but we did not find them in the genome of the strain RZME27^T^ as also occurred in *P. pelagicum* [11]. However, several genes involved in colonization were found in the genome of strain RZME27^T^, such as those related with the type IV secretion system, located in a genomic island, which is widespread among endophytic bacteria [51,52]. We also identified genes involved in motility, such as those codifying flagellar biosynthesis proteins, located in a genome region, which also contains chemotaxis involved genes (*che*) in neighbour positions regulated by *luxR* genes. The presence of *luxR* genes, which are associated to chemotaxis, as well as flagellar proteins indicates that these functions are modulated by population density where quorum sensing response plays the main role [53]. The genome of strain RZME27^T^ and those of its closest relatives, *N. galegae*, *P. pelagicum* and *R. leguminosarum*, also contain the *luxI* gene, found by manual mining and antiSMASH analysis, which encodes a homoserine lactone synthase, commonly found in Proteobacteria and necessary for the production of homoserine lactone-quorum sensing related molecules in these bacteria [54].

Several genes found in the strain RZME27^T^ involved in exopolysaccharide biosynthesis and degradative hydrolytic enzymes production may also play a role in the establishment of bacteria in rhizosphere and endosphere environments, as has been reported for rhizobia and other bacterial endophytes [55,56]. Particularly, genes involved in cellulose biosynthesis and cellulase production found in the genomes of the strain RZME27^T^ and the type strains of *N. galegae*, *P. pelagicum* and *R. leguminosarum* were similar to those of *celABC* operon of *Rhizobium* strains nodulating clover [57]. Remarkably, annotations showed the presence of genes involved the synthesis of glycine-betaine, one of the main osmoprotectant molecules in bacteria [58,59], in the genomes of the strain RZME27^T^ and its closest relatives *N. galegae* and *P. pelagicum*. The results obtained in the in vitro phenotypic tests confirmed that the strain RZME27^T^ can grow under saline osmotic stress, as well as *N. galegae*, which also grows in these conditions [60], and as *P. pelagicum*, which was originally isolated from a saline environment [11].

Comparative analysis helps to understand relationships between different species, and their evolution and genomic adaptation throughout the identification of clusters of orthologous genes originated by vertical inheritance from a unique common ancestor [61,62]. The highest number of clusters was found in the pangenome of the type strains of *R. leguminosarum* and *N. galegae*, which are able to nodulate legumes, confirming that symbiotic rhizobia have very complex pangenomes and also these two strains shared the highest number of clusters (Figure 4B). The strains sharing the highest number of clusters were the type strains of *N. galegae* and *R. leguminosarum*, all of them isolated from plant sources (Figure 4B). However, *P. pelagicum*, which was isolated from a marine habitat, shares less protein clusters with the species inhabiting soil related niches, than these species among them (Figure 4B). Remarkably, the strain RZME27^T^ has a higher number of unshared clusters with the type species of its related genera than these type species among themselves. Nevertheless, the total number of clusters shared by the analyzed strains was quite high taking into account their different ecological niches.

## 5. Conclusions

In this work, we reported the genomic and phenotypic characteristics of a strain named RZME27^T^ isolated from *Zea mays* roots growing in a field located at León (Northern Spain). The results of 16S rRNA gene and genome analyses showed that this strain belongs to the family *Rhizobiaceae* with its closest related taxa belonging to genera *Neorhizobium*, *Rhizobium* and *Pseudorhizobium*. The genes involved in legume nodulation and nitrogen fixation were absent in the genome of the strain RZME27^T^; nevertheless, genes involved in plant colonization and genes involved in osmotolerance were found. The comparative pan-genomic analysis showed that 78 protein clusters present in the strain RZME27^T^ were not found in the type strains of its closest relatives *N. galegae* and *P. pelagicum*, nor in the type species of the genus *Rhizobium*. The ANIb values between the genomes of the strain RZME27^T^ and those the type species of *Neorhizobium* and *Pseudorhizobium* were similar or lower than those found between the genomes other genera from the family *Rhizobiaceae* recently described, supporting the affiliation of the strain RZME27^T^ to a novel genus for which we propose the name *Endobacterium cerealis* gen. nov. sp. nov.

## 6. Description of *Endobacterium* gen. nov.

*Endobacterium* (En.do.bac’te.ri.um. Gr. pref. *endo*, within; L. neut. n. *bacterium*, a small rod; N.L. masc. n. *Endobacterium*, a rod isolated from the endopshere of *Zea mays*).

Aerobic, Gram negative, motile and non-sporulated rods forming white colonies on YMA. Optimal growth at 28 °C and pH 7. Catalase and oxidase were positive. Nitrate reduction is positive. Glucose fermentation is negative. Esculin hydrolysis and production of urease and β-galactosidase were positive. Production of indole, H_2_S and arginine dehydrolase is negative. Growth was observed up to 4% (w/v) NaCl. The main fatty acids are C_18:1_ω7c/C_18:1_ω6c. The G+C content of genomic DNA of the type strain of the type species is 58.8 mol%. Delineation of the genus was determined by the phylogenetic information from 16S rRNA gene sequence and by the analysis of the complete genome sequence. The type species is *Endobacterium cerealis*.

## 7. Description of *Endobacterium cerealis* sp. nov.

*Endobacterium cerealis* (ce.re.al‘is. N.L. masc. adj. cerealis, of or pertaining to cereal, because the type strain was isolated from the cereal *Zea mays*).

Gram-stain negative rods that are 0.7–0.8 μm wide and 2.5–3.0 μm long. They are motile by means of one polar flagellum. Colonies are small, pearl white colored in YMA at 28 °C, which is the optimal growth temperature. The optimal pH for growth is 7–7.5 and the optimal NaCl concentration is 1%. Growth was observed in presence of 3.5% (*w/v*) NaCl and it was weak in presence of 4% (*w/v*) NaCl. Growth was observed in a range from 15 to 37 °C and pH from 6 to 8. No growth observed at 40 °C or pH 5. Oxidase and catalase positive. Nitrate reduction is positive. Glucose fermentation is negative. Esculin hydrolysis and production of urease and β-galactosidase were positive. Production of indole, H_2_S and arginine dehydrolase is negative. Assimilation as sole carbon and energy source of d-glucose, d-mannose, l-rhamnose, *N*-acetyl-glucosamine, l-ribose, inositol, sucrose, maltose, mannitol, melibiose, l-fucose, l-sorbose, l-arabinose, d, l-lactate, gluconate, malate, l-alanine, 5 keto-gluconate, l-histidine and l-proline is positive. Assimilation of itaconate, suberate, malonate, glycogen, 3-hydroxybenzoate, salicine, propionate, caprate, acetate, valerate, citrate, adipate, phenylacetate, 2 keto-gluconate, l-serine, 4-hydroxybenzoate and 3-hydroxybutyrate is negative. The fatty acid profile consists of C_18:1_ ω6*c*/C_18:1_ ω7*c* (summed feature 8), C_16:0_, C_16:0_ 3OH, C_18:0_ 3OH, C_16:1_ 6c/C_16:1_ ω7c (summed feature 3), C_14:0_ 3OH/C_16:1_ iso I (summed feature 2), C_8:1_ ω7c 11-methyl, C_17:0_ and C_18:0_. The G+C content of the strain RZME27^T^ is 58.8 mol%.

The type strain RZME27^T^ (= LMG 31256^T^ = CECT 9794^T^) was isolated from roots of *Zea mays* in Spain.

## Figures and Tables

**Table 1:** Genome properties of the *Endobacterium cerealis*, its closest related genera and the type species of the type genus of family *Rhizobiaceae*.

Attributes	*E. cerealis* RZME27^T^	*P. pelagicum* RI-200B4^T^	*N. galegae* HAMBI 540^T^	*R. leguminosarum* USDA 2370^T^
Sequence size (bp)	6,114,623	5,134,606	6,455,027	7,851,935
Number of contigs	51	62	2	108
GC content (%)	58.8	62.8	61.3	60.6
Longest contig size	649,048	392,382	4,647,962	1,820,721
Shortest contig size	285	4118	1,807,065	502
N50 value	345,671	184,300	4,647,962	413,138
L50 value	7	11	1	5
Total number of genes	5893	4509	6248	7753
Total CDSs	5836	4458	6,181	7699
Number of coding sequences/CDSs (with protein)	5705	4417	6014	7312
Pseudogenes	131	41	167	387
Number of RNAs (tRNAs, rRNAs, ncRNAs)	57 (49, 4, 4)	51 (47, 3, 1)	67 (51, 9, 7)	54 (46, 4, 4)

**Table 2:** Calculated ANIb values for available genomes of the type strains from the type species of the genera included in the family *Rhizobiaceae* (the accession numbers for these genomes are in parentheses). *Endobacterium cerealis* RZME27^T^ (WIXI00000000), *Neorhizobium galegae* HAMBI 540^T^ (HG938353.1), *Pseudorhizobium pelagicum* R1-200B4^T^ (JOKI01000001.1), *Rhizobium leguminosarum* USDA 2370^T^ (QBLB00000000.1), *Georhizobium profundi* WS11^T^ (CP032509.1), *Gellertiella. hungarica* DSM 29853^T^ (IMG Ga0373349), *Ciceribacter lividus* DSM 25528^T^ (QPIX00000000.1), *Shinella granuli* DSM 18401^T^ (SLVX00000000.1), *Ensifer adhaerens* Casida A^T^ (CP015880.1), *Allorhizobium undicola* ORS 992^T^ (JHXQ00000000.1), *Agrobacterium radiobacter* LMG 140^T^ (MRDG01000013.1), *Pararhizobium giardinii* H152^T^ (ARBG00000000.1).

Species	RZME27^T^	HAMBI 540^T^	R1-200B4^T^	USDA 2370^T^	WS11^T^	DSM 29853^T^	DSM 25528^T^	DSM 18401^T^	Casida A^T^	ORS 992^T^	LMG 140^T^	H152^T^
RZME17^T^	100											
HAMBI 540^T^	75.61	100										
R1-200B4^T^	75.09	75.40	100									
USDA 2370^T^	73.34	74.49	73.03	100								
WS11^T^	70.91	70.23	70.69	70.24	100							
DSM 29853^T^	72.78	72.34	73.02	72.56	70.75	100						
DSM 25528^T^	73.82	73.47	74.19	73.15	70.87	73.33	100					
DSM 18401^T^	73.14	73.53	73.40	73.92	71.08	73.55	73.70	100				
Casida A^T^	72.75	73.20	73.03	73.64	70.87	72.82	73.04	76.03	100			
ORS 992^T^	72.97	72.70	72.65	72.81	69.94	72.50	73.27	72.75	72.23	100		
DSM 30147^T^	74.30	73.77	73.39	73.29	70.10	72.26	73.11	73.20	72.63	72.76	100	
H152^T^	72.90	73.17	72.73	74.57	70.70	72.57	72.97	75.43	75.74	71.96	72.52	100

**Table 3:** Cellular fatty acid composition of *Endobacterium cerealis*, its closest related genera and the type species of the type genus of family *Rhizobiaceae*.

Fatty Acid *	*E. cerealis* RZME27^T^	*N. galegae* HAMBI 540^T^	*P. pelagicum* R1-200B4^T^	*R. leguminosarum* USDA 2370^T^
C_16:0_	8.86	11.58	7.69	7.18
C_17:0_	0.10	1.91	nd	nd
C_18:0_	2.08	2.28	2.26	12.71
C_16:0_ 3OH	2.14	2.54	nd	nd
C_18:0_ 3OH	1.30	1.60	1.49	0.99
C_18:1_ω7c 11-methyl	0.38	0.55	3.04	7.06
C_19:0_ cyclo ω8c	nd	10.76	4.37	3.74
Summed feature 2 ^1^	5.18	4.63	4.41	3.59
Summed feature 3 ^2^	1.78	0.63	1.49	1.19
Summed feature 8 ^3^	77.70	61.14	73.84	62.77

^1^ Summed feature 2: (C_14:0_ 3OH/C_16:1_ iso I/C_12:0_ aldehyde); ^2^ Summed feature 3: (C_16:1_ω7c/C_16:1_ω6c);^3^ Summed feature 8: (C_18:1_ω7c/C_18:1_ω6c); * Fatty acids present in amounts lower than 1% in all species are not shown.

**Table 4:** Differential phenotypic characteristics of *Endobacterium cerealis*, its closest related genera and the type species of the type genus of family *Rhizobiaceae*.

Characteristics	*E. cerealis* RZME27^T^	*N. galegae* HAMBI 540^T^	*P. pelagicum* R1-200B4^T^	*R. leguminosarum* USDA 2370^T^
Colony colour (on YMA)	white	white-pink	white	white-cream
Nitrate reduction	+	−	+	−
Growth in 1% NaCl	+	+	+	−
Growth in 4% NaCl	+	−	+	−
Growth in 7% NaCl	−	−	+	−
Growth at 37 °C	w	+	+	−
Assimilation of (API 20NE):				
Malate	+	+	−	w
Assimilation of (API 32GN):				
l-rhamnose	+	−	+	+
d-ribose	w	+	−	+
Inositol	+	+	−	+
Melibiose	+	+	−	+
5-keto-gluconate	+	+	+	−
2-keto-gluconate	−	−	w	−
Propionate	+	−	−	−
3-hydroxybutyrate	−	−	−	+
l-histidine	+	+	−	+
l-alanine	w	+	w	−
l-serine	−	−	+	−

+: positive, −: negative, w: weak.

## Supplementary Materials

The following are available online at https://www.mdpi.com/2076-2607/8/6/939/s1.
