# Supplementary material for: Genome Analysis of Endobacterium cerealis, a Novel Genus and Species Isolated from Zea mays Roots in North Spain
Source: Microorganisms. 2020 Jun 22;8(6):939. doi: 10.3390/microorganisms8060939 (PMC7356062; doi:10.3390/microorganisms8060939)
Supplement: Supplementary file 1 [file microorganisms-08-00939-s001.zip › Suplementary Files/Suplementary Tables.docx]

| **Species name** | **Strain** | **Accession number** | **BioProject** | **BioSample** |
| --- | --- | --- | --- | --- |
| *Endobacterium* *cerealis* | RZME27^T^ | WIXI00000000 | PRJNA576662 | SAMN13000627 |
| *Neorhizobium* *galegae* | HAMBI 540^T^ | HG938353 | PRJEB1950 | SAMEA3139073 |
| *Pseudorhizobium* *pelagicum* | R1-200B4^T^ | JOKI00000000 | PRJNA252589 | SAMN02864219 |
| *Rhizobium* *leguminosarum* | USDA2370^T^ | QBLB00000000 | PRJNA450070 | SAMN08929321 |
| *Georhizobium profundi* | WS11^T^ | CP032509 | PRJNA492217 | SAMN10095205 |
| *Gellertiella. hungarica* | DSM 29853^T^ | IMG Ga0373349 | PRJNA583282 | SAMN13172762 |
| *Ciceribacter lividus* | DSM 25528^T^ | QPIX00000000 | PRJNA463375 | SAMN09074871 |
| *Shinella granuli* | DSM 18401^T^ | SLVX00000000 | PRJNA520307 | SAMN10866395 |
| *Ensifer adhaerens* | Casida A^T^ | CP015880 | PRJNA247817 | SAMN02781308 |
| *Allorhizobium undicola* | ORS 992^T^ | JHXQ00000000 | PRJNA223117 | SAMN02744004 |
| *Agrobacterium radiobacter* | LMG 140^T^ | MRDG01000013 | PRJNA355337 | SAMN06074341 |
| *Pararhizobium giardinii* | H152^T^ | ARBG00000000 | PRJNA165331 | SAMN02256420 |

**Table S1.** Data regarding to the genomes of the type strains used in this study.

**Table S2**. Similarity values for the 16S rRNA gene sequences of the type strains from the type species of the genera included in the Family *Rhizobiaceae. Endobacterium cerealis* RZME27^T^ (WIXI00000000), *Neorhizobium galegae* HAMBI 540^T^ (HG938353.1), *Pseudorhizobium pelagicum* R1-200B4^T^ (JOKI01000001.1), *Rhizobium leguminosarum* USDA 2370^T^ (NZ_QBLB00000000.1), *Georhizobium profundi* WS11^T^ (CP032509.1), *Gellertiella. hungarica* DSM 29853^T^ (IMG Ga0373349), *Ciceribacter lividus* DSM 25528^T^ (NZ_QPIX00000000.1), *Shinella granuli* DSM 18401^T^ (NZ_SLVX00000000.1), *Ensifer adhaerens* Casida A^T^ (CP015880.1), *Allorhizobium undicola* ORS 992^T^ (NZ_JHXQ00000000.1), *Agrobacterium radiobacter* LMG 140^T^ (MRDG01000013.1), *Pararhizobium giardinii* H152^T^ (NZ_ARBG00000000.1).

| **Species** | **RZME27^T^** | **HAMBI 540^T^** | **R1-200B4^T^** | **USDA 2370^T^** | **WS11^T^** | **DSM 29853^T^** | **DSM 25528^T^** | **DSM 18401^T^** | **Casida A^T^** | **ORS 992^T^** | **LMG 140^T^** | **H152^T^** |
| --- | --- | --- | --- | --- | --- | --- | --- | --- | --- | --- | --- | --- |
| RZME17^T^ | 100 |  |  |  |  |  |  |  |  |  |  |  |
| HAMBI 540^T^ | 97.4 | 100 |  |  |  |  |  |  |  |  |  |  |
| R1-200B4^T^ | 95.4 | 97.1 | 100 |  |  |  |  |  |  |  |  |  |
| USDA 2370^T^ | 95.8 | 95.9 | 95.3 | 100 |  |  |  |  |  |  |  |  |
| WS11^T^ | 94.3 | 93.7 | 93.8 | 93.2 | 100 |  |  |  |  |  |  |  |
| DSM 29853^T^ | 94.6 | 95.0 | 94.5 | 95.0 | 95.1 | 100 |  |  |  |  |  |  |
| DSM 25528^T^ | 95.0 | 95.3 | 93.4 | 94.4 | 94.8 | 96.9 | 100 |  |  |  |  |  |
| DSM 18401^T^ | 94.9 | 94.2 | 93.4 | 93.7 | 95.8 | 96.0 | 95.6 | 100 |  |  |  |  |
| Casida A^T^ | 95.0 | 94.7 | 94.0 | 95.5 | 95.8 | 97.6 | 97.7 | 95.9 | 100 |  |  |  |
| ORS 992^T^ | 94.9 | 94.7 | 93.4 | 93.2 | 93.7 | 93.1 | 94.0 | 93.8 | 93.9 | 100 |  |  |
| LMG 140^T^ | 95.5 | 95.3 | 94.1 | 94.7 | 95.3 | 95.8 | 96.4 | 95.3 | 96.3 | 94.6 | 100 |  |
| H152^T^ | 95.2 | 95.9 | 94.3 | 96.2 | 95.0 | 96.2 | 96.6 | 95.3 | 97.5 | 93.9 | 96.5 | 100 |

**Table S3.** Subsystems of genes according to SEED database (RAST server).

| **Subsystems** | **Number of genes** |
| --- | --- |
| Cofactors, Vitamins, Prosthetic Groups Pigments | 152 |
| Cell Wall and Capsule | 35 |
| Virulence, Disease and Defense | 36 |
| Potassium metabolism | 12 |
| Miscellaneous | 46 |
| Phages, Prophages, Transposable elements, Plasmids | 15 |
| Membrane Transport | 88 |
| Iron acquisition and metabolism | 38 |
| RNA Metabolism | 44 |
| Nucleosides and Nucleotides | 95 |
| Protein Metabolism | 199 |
| Motility and Chemotaxis | 56 |
| Regulation and Cell signaling | 40 |
| Secondary Metabolism | 4 |
| DNA Metabolism | 115 |
| Fatty Acids, Lipids, and Isoprenoids | 53 |
| Nitrogen Metabolism | 25 |
| Dormancy and Sporulation | 1 |
| Respiration | 114 |
| Stress Response | 92 |
| Metabolism of Aromatic Compounds | 26 |
| Amino Acids and Derivatives | 294 |
| Sulfur Metabolism | 8 |
| Phosphorus Metabolism | 28 |
| Carbohydrates | 313 |

**Table S4.** Protein annotation via dbCAN2 meta server, using DIAMOND, HMMER and HotPep via CAZy, dbCAN and PPR, respectively. The combination of these 3 tools provides the full annotation of carbohydrate-related enzymes. Tools information according to Zhang et al 2018 [39] are: DIAMOND (E-Value < 1e-102), HMMER (E-value < 1e-15, coverage > 0.35) and HotPep (Frequency > 2.6, Hits > 6).

| **CAZyme type** | **Total** | **DIAMOND** | **HMMER** | **HotPep** |
| --- | --- | --- | --- | --- |
| Glycosil Hydrolases (GH) | 73 | 64 | 54 | 53 |
| Glycosil Transferases (GT) | 60 | 50 | 45 | 35 |
| Carbohydrate-binding Module (CBM) | 9 | 5 | 9 | 6 |
| Carbohydrate Esterase (CE) | 12 | 4 | 10 | 5 |
| Auxiliary Activities (AA) | 13 | 4 | 12 | 10 |
| Polysaccharide Lyase (PL) | 3 | 3 | 2 | 3 |
| **Total** | 170 | 130 | 132 | 112 |

**Table S5.** KEGG annotation via KofamKOALA of proteins of the strain RZME27^T^ genomes. Reconstruction of complete and incomplete metabolic pathways according to the KEGG mapper.

| **Pathway modules** | **Total** | **Number of genes related to** | | |
| --- | --- | --- | --- | --- |
|  |  | **Complete pathways** | | **Incomplete pathways** |
| **Carbohydrate metabolism** |  | |  |  |
| Central carbohydrate metabolism | 86 | | 76 | 10 |
| Other carbohydrate metabolism | 74 | | 40 | 34 |
| **Energy metabolism** | | | | |
| Carbon fixation | 59 | | 4 | 55 |
| Methane metabolism | 8 | | 0 | 8 |
| Nitrogen metabolism | 6 | | 0 | 6 |
| Sulfur metabolism | 6 | | 6 | 0 |
| ATP synthesis | 50 | | 40 | 10 |
| **Lipid metabolism** | | | | |
| Fatty acid metabolism | 20 | | 14 | 6 |
| Lipid metabolism | 7 | | 4 | 3 |
| **Nucleotide metabolism** | | | | |
| Purine metabolism | 28 | | 22 | 6 |
| Pyrimidine metabolism | 17 | | 3 | 14 |
| **Amino acid metabolism** | | | | |
| Serine and threonine metabolism | 12 | | 8 | 2 |
| Cysteine and methionine metabolism | 14 | | 2 | 12 |
| Branched-chain amino acid metabolism | 17 | | 4 | 13 |
| Lysine metabolism | 24 | | 9 | 15 |
| Arginine and proline metabolism | 20 | | 11 | 9 |
| Polyamine biosynthesis | 6 | | 2 | 4 |
| Histidine metabolism | 14 | | 5 | 9 |
| Aromatic amino acid metabolism | 19 | | 13 | 6 |
| Other amino acid metabolism | 4 | | 2 | 2 |
| **Glycan metabolism** | | | | |
| Lipopolysaccharide metabolism | 20 | | 0 | 20 |
| **Metabolism of cofactors and vitamins** | | | | |
| Cofactor and vitamin metabolism | 89 | | 20 | 69 |
| **Biosynthesis of terpenoids and polyketides** | | | | |
| Terpenoid backbone biosynthesis | 10 | | 0 | 10 |
| Polyketide sugar unit biosynthesis | 4 | | 4 | 0 |
| **Biosynthesis of other secondary metabolites** | | | | |
| Biosynthesis of other secondary metabolites | 2 | | 0 | 2 |
| **Xenobiotics biodegradation** | | | | |
| Aromatics degradation | 6 | | 0 | 6 |
| **Others** | | | | |
| Pathogenicity | 5 | | 0 | 5 |
| Drug resistance | 8 | | 0 | 8 |
| Plant pathogenicity | 6 | | 0 | 6 |
