# Supplementary figures and images for: Genome Analysis of Endobacterium cerealis, a Novel Genus and Species Isolated from Zea mays Roots in North Spain
Source: Microorganisms. 2020 Jun 22;8(6):939. doi: 10.3390/microorganisms8060939 (PMC7356062; doi:10.3390/microorganisms8060939)

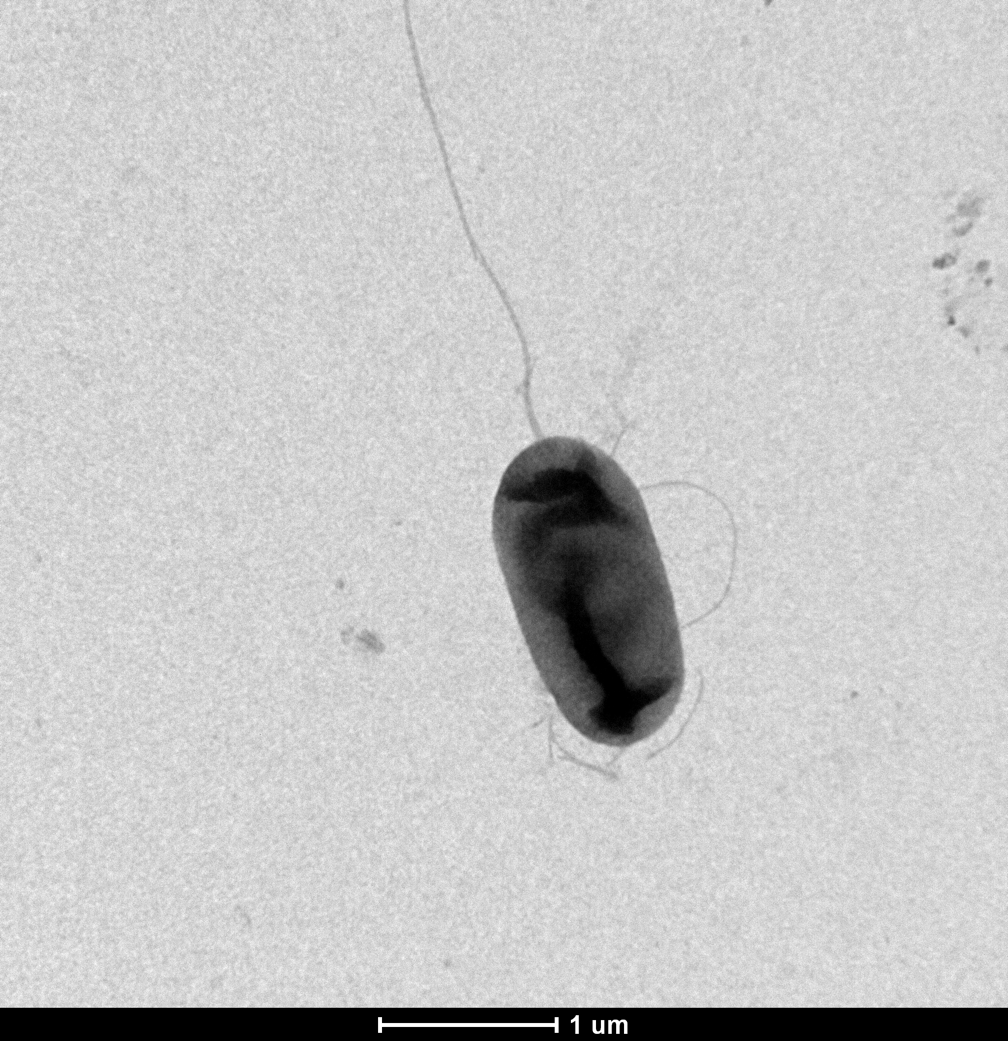


**Figure S1.** Electron micrograph of *Endobacterium cerealis* RZME27T showing the polar flagellum

Supplement: Supplementary file 1 [file microorganisms-08-00939-s001.zip › Suplementary Files/Supplementary Figure.docx]
